# Tumor immunology: unraveling the complex interaction between tumors and the immune system: a narrative review

**DOI:** 10.1097/MS9.0000000000003719

**Published:** 2025-08-12

**Authors:** Emmanuel Ifeanyi Obeagu

**Affiliations:** Department of Biomedical and Laboratory Science, Africa University, Zimbabwe

**Keywords:** cancer immunotherapy, CAR-T cell therapy, immune checkpoints, immune evasion, tumor microenvironment (TME)

## Abstract

Tumor immunology is a critical area of cancer research that focuses on the interplay between the immune system and tumor cells. The immune system has the ability to recognize and eliminate cancer cells through various immune cells, such as cytotoxic T lymphocytes (CTLs) and natural killer (NK) cells. However, tumors have developed sophisticated mechanisms to evade immune detection, such as the loss of tumor antigen expression, recruitment of immunosuppressive cells, and upregulation of immune checkpoints. These immune evasion strategies allow tumors to grow uncontrollably and metastasize. Immunotherapy has emerged as a promising treatment modality that harnesses the power of the immune system to fight cancer. Key strategies include immune checkpoint inhibitors, which block inhibitory receptors like programmed cell death protein 1 and cytotoxic T-lymphocyte-associated protein 4, and adoptive cell therapies, which involve the transfer of tumor-specific T cells or NK cells into patients. Additionally, monoclonal antibodies and cancer vaccines are being explored to directly target tumor cells and enhance immune responses. Despite the success of these therapies, challenges such as tumor heterogeneity, resistance to treatment, and immune-related adverse effects persist.

## Introduction

Cancer remains one of the leading causes of morbidity and mortality worldwide, with an estimated 20 million new cases and 10 million deaths globally in 2024 alone. While significant strides have been made in early detection, molecular diagnostics, and targeted therapies, many cancers continue to evade treatment due to their ability to subvert host immune responses. This dynamic interaction between tumors and the immune system has given rise to the field of tumor immunology, which seeks to understand the dual role of immunity in both suppressing and facilitating cancer progression^[1,2]^. The immune system has an intrinsic capacity to recognize and eliminate transformed cells through processes collectively known as immunosurveillance. Key immune effectors – including cytotoxic T lymphocytes, natural killer (NK) cells, and antigen-presenting cells (APCs) – can detect tumor-associated antigens (TAAs) and initiate targeted immune responses. However, tumors often develop mechanisms to escape these defenses, either through the selection of less immunogenic variants (immunoediting) or by creating an immunosuppressive microenvironment that impairs immune activation[3]. One of the hallmarks of cancer is immune evasion, which is orchestrated by a complex network of cellular and molecular mechanisms. Tumors can downregulate major histocompatibility complex (MHC) molecules, secrete inhibitory cytokines such as TGF-β and IL-10, and recruit regulatory immune cells, including Tregs and myeloid-derived suppressor cells (MDSCs). These strategies collectively dampen anti-tumor immunity and promote tumor tolerance, enabling unchecked growth and metastasis^[4–6]^.

**HIGHLIGHTS**

- Tumors evade immune detection through immune checkpoint pathways like programmed cell death protein 1/programmed cell death-ligand 1.
- Tumor microenvironment suppresses immune responses, promoting cancer progression.
- Immunotherapies, including chimeric antigen receptor-T cells, enhance anti-tumor immunity.
- Neoantigen presentation triggers targeted immune activation.
- Regulatory T cells and myeloid-derived suppressor cells weaken immune defense.

Over the past two decades, immunotherapy has revolutionized cancer treatment by harnessing the immune system to target malignant cells. Immune checkpoint inhibitors, such as those targeting programmed cell death protein 1 (PD-1)/programmed cell death-ligand 1 (PD-L1) and cytotoxic T-lymphocyte-associated protein 4 (CTLA-4), have demonstrated remarkable clinical efficacy in cancers such as melanoma, non-small cell lung cancer (NSCLC), and renal cell carcinoma. Despite these advances, not all patients respond to immunotherapy, and resistance – both primary and acquired – remains a major clinical challenge^[7–10]^. To address these limitations, research has increasingly focused on combination therapies that integrate immunotherapy with chemotherapy, radiation, targeted agents, or oncolytic viruses. Furthermore, there is growing interest in identifying and targeting specific immunosuppressive pathways, such as the nuclear factor kappa-light-chain-enhancer of activated B cells (NF-κB) signaling cascade, which plays a critical role in tumor-associated inflammation and immune modulation^[11–13]^. This review aims to provide a comprehensive yet focused narrative on the intricate interplay between tumors and the immune system. It explores the mechanisms of immune recognition, tumor-induced immune suppression, recent advances in immunotherapy, and emerging strategies to overcome therapeutic resistance. Special attention is given to the role of the tumor microenvironment (TME), key molecular regulators, and combination strategies aimed at enhancing immune responses.

### Aim

The aim of this review is to provide an in-depth exploration of the evolving field of cancer immunology, highlighting the mechanisms through which the immune system interacts with cancer cells and the challenges that arise from tumor immune evasion.

### Justification of the review

The evolving field of tumor immunology has significantly transformed our understanding of cancer biology, offering new perspectives on disease progression, immune surveillance, and therapeutic innovation. Despite the remarkable clinical success of immunotherapeutic agents – such as immune checkpoint inhibitors – their efficacy remains limited to a subset of patients, and resistance mechanisms continue to emerge. As a result, there is a critical need to comprehensively synthesize the multifaceted interactions between tumors and the immune system to better understand the determinants of therapeutic response and failure^[14,15]^. While numerous reviews have addressed individual components of tumor immunology, many have focused narrowly on specific pathways, immune cell subsets, or clinical trials. This fragmented approach often overlooks the complex, dynamic, and bidirectional nature of tumor-immune interactions that underpin cancer immunoediting, immune evasion, and the establishment of an immunosuppressive microenvironment. Furthermore, the integration of emerging immunosuppressive mechanisms – such as NF-κB signaling – and their therapeutic targeting is underrepresented in existing literature[16]. This narrative review seeks to bridge this gap by providing an integrated and accessible overview of the core mechanisms governing tumor immunity, including immune recognition, immunoediting, immune escape, and immunotherapy resistance. It also expands on current combination strategies and promising molecular targets that modulate the immune landscape of tumors. By synthesizing both foundational knowledge and contemporary advances, the review offers a holistic framework that is valuable for researchers, clinicians, and students engaged in cancer immunology and oncology.

## Methods

This narrative review was conducted to synthesize current knowledge on the interplay between the immune system and tumor biology, with a focus on immune surveillance, evasion mechanisms, and immunotherapeutic strategies. Unlike systematic reviews, the narrative approach was chosen to allow a broad and integrative analysis of complex and evolving themes in tumor immunology. A comprehensive literature search was performed across major electronic databases, including PubMed, Scopus, and Web of Science, covering publications up to June 2025. The search strategy employed combinations of keywords and Medical Subject Headings such as *“tumor immunology,” “immune evasion,” “tumor microenvironment,” “immune checkpoint inhibitors,” “NF-κB in cancer,”* and *“cancer immunotherapy.”* Articles were filtered to include original research, clinical studies, systematic reviews, meta-analyses, and high-impact narrative reviews published in English.

The inclusion criteria prioritized peer-reviewed publications that provided mechanistic insights, preclinical or clinical data, or therapeutic perspectives relevant to tumor-immune interactions. Studies focusing on major cancer types (e.g. breast, lung, cervical, and melanoma) were given particular attention due to the availability of immunotherapy data. Key publications from leading journals in immunology, oncology, and translational medicine were selected to ensure scientific rigor and relevance. Data were manually extracted and synthesized thematically. Topics were grouped into key domains: immune recognition of tumors, immune evasion and suppression, immune checkpoint pathways, immunotherapeutic interventions, and emerging molecular targets such as NF-κB. The quality and relevance of sources were assessed based on journal impact, citation frequency, and contribution to current understanding. Although this review does not follow PRISMA guidelines due to its narrative nature, it adheres to scholarly standards of transparency, comprehensiveness, and critical appraisal. The synthesis emphasizes both well-established principles and emerging areas of interest, with the aim of providing a cohesive and insightful contribution to the field of tumor immunology.

### The immune system and cancer surveillance

The immune system plays a crucial role in maintaining tissue homeostasis and protecting the host from malignant transformation. The concept of cancer immunosurveillance posits that immune cells can detect and eliminate nascent tumor cells before they develop into clinically apparent malignancies. This process is mediated primarily by components of both the innate and adaptive immune systems, which recognize TAAs or stress-induced molecules on transformed cells[16]. Key players in immune surveillance include NK cells, dendritic cells (DCs), cytotoxic CD8+ T lymphocytes, and helper CD4+ T cells. NK cells are equipped to eliminate cells with downregulated MHC class I molecules, a common feature of tumor cells attempting to evade T cell detection. DCs function as potent APCs that capture TAAs and migrate to lymphoid organs to prime naive T cells, bridging the innate and adaptive immune responses. Activated CD8+ T cells then home to tumor sites and mediate cytotoxicity through perforin, granzymes, and the Fas-FasL pathway[17].

**Figure 1.**
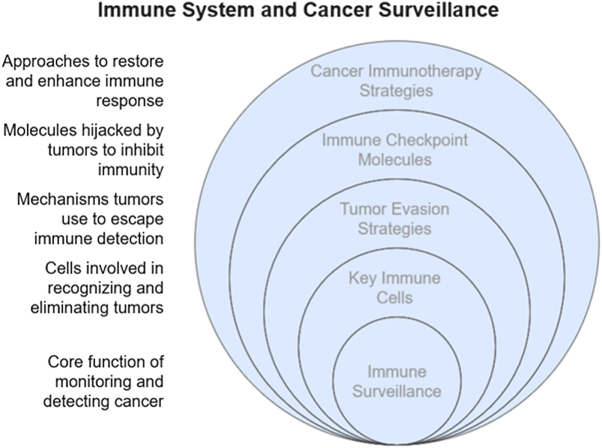
Immune system and cancer surveillance.

The process of immunosurveillance is not infallible. Tumor cells can undergo immunoediting, a dynamic process encompassing three phases: elimination, equilibrium, and escape. In the elimination phase, the immune system successfully destroys most transformed cells. During the equilibrium phase, selective immune pressure allows for the survival of variants with reduced immunogenicity. Ultimately, in the escape phase, these tumor cell variants establish a clinically detectable malignancy by evading immune detection or actively suppressing immune responses[18]. Recent evidence has confirmed the clinical relevance of cancer immunosurveillance. For instance, cancer incidence is significantly higher in immunocompromised individuals, such as organ transplant recipients or patients with HIV/AIDS. Moreover, tumor-infiltrating lymphocytes (TILs) have been associated with improved prognosis in several cancer types, highlighting the beneficial role of the immune response in controlling tumor growth[19]. Importantly, immune surveillance is context-dependent and can be influenced by factors such as the TME, systemic inflammation, and host genetics. The TME, often characterized by hypoxia, metabolic dysregulation, and chronic inflammation, can modulate immune cell function and either promote or hinder immune surveillance (Fig. 1)[20].

### Mechanisms of tumor immune evasion

Despite the immune system’s inherent capacity to detect and eliminate malignant cells, tumors frequently develop sophisticated strategies to evade immune destruction. These immune escape mechanisms are critical for tumor progression and metastasis and represent major obstacles to effective immunotherapy. Tumor immune evasion can be broadly categorized into three major domains: reduced immunogenicity, active immunosuppression, and altered immune cell recruitment[21].

#### Antigen modulation and loss of immunogenicity

Tumor cells can downregulate or alter the expression of TAAs and MHC molecules, particularly MHC class I. This impairs antigen presentation and prevents effective recognition by cytotoxic CD8+ T lymphocytes. Additionally, tumors may undergo epigenetic modifications that silence genes encoding TAAs, further reducing immune visibility. These changes enable malignant cells to escape T cell-mediated cytotoxicity and persist undetected within the host^[22,23]^.

#### Secretion of immunosuppressive cytokines and factors

Many tumors actively secrete immunosuppressive molecules such as transforming growth factor-beta (TGF-β), interleukin-10 (IL-10), and vascular endothelial growth factor (VEGF). These cytokines suppress DC maturation, impair T cell activation, and promote regulatory T cell (Treg) expansion. Moreover, VEGF contributes to abnormal angiogenesis and restricts the infiltration of effector immune cells into the TME. Through these mechanisms, tumors create a milieu that favors immune tolerance over immune rejection^[24,25]^.

#### Recruitment and expansion of immunoregulatory cells

Tumors can recruit immunosuppressive cell populations – including Tregs, MDSCs, and tumor-associated macrophages (TAMs) – to inhibit effector T cell function and sustain immune evasion. Tregs suppress immune responses through cell–cell contact and cytokine secretion, while MDSCs inhibit T cell proliferation via the production of nitric oxide, reactive oxygen species, and arginase-1. TAMs, particularly those polarized toward the M2 phenotype, release anti-inflammatory cytokines and facilitate tumor growth, invasion, and metastasis[26].

#### Upregulation of immune checkpoint molecules

Tumors frequently exploit immune checkpoint pathways such as PD-1/PD-L1 and CTLA-4 to suppress T cell activation and induce functional exhaustion of TILs. PD-L1 expression on tumor or stromal cells interacts with PD-1 receptors on T cells, leading to T cell anergy and apoptosis. This checkpoint-mediated inhibition is a major target for current immunotherapies, underscoring its central role in immune escape[27].

#### Modulation of metabolic pathways

The metabolic landscape of the TME further contributes to immune evasion. Hypoxia, acidosis, and nutrient depletion (e.g. glucose and tryptophan) impair the function of effector immune cells. For instance, the enzyme indoleamine 2,3-dioxygenase, often upregulated in tumors, depletes tryptophan and generates immunosuppressive metabolites that inhibit T cell proliferation and promote Treg development[28].

#### Resistance to apoptosis and immune cytotoxicity

Some tumors acquire resistance to immune-mediated cell death through the overexpression of anti-apoptotic proteins such as Bcl-2 and FLIP, or by mutating components of the death receptor pathway. Additionally, tumor cells may shed decoy receptors or express surface molecules that interfere with immune synapse formation, thus preventing effective immune attack[29].

### Immune checkpoints in cancer

**Figure 2.**
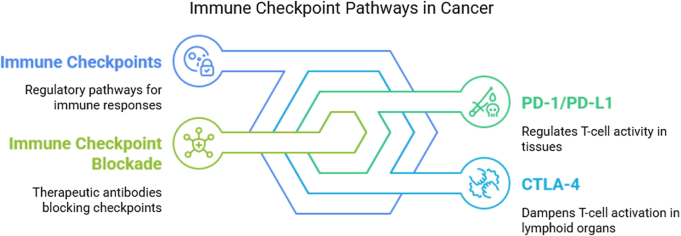
Immune checkpoint pathways.

Immune checkpoints are a cornerstone of modern immunotherapy. While the PD-1/PD-L1 and CTLA-4 pathways have been most extensively studied, recent evidence highlights the roles of other inhibitory receptors such as LAG-3, TIM-3, and TIGIT. Therapies targeting these novel checkpoints are in various stages of clinical trials and hold promise for patients who do not respond to conventional checkpoint inhibitors. For instance, LAG-3 inhibitors like relatlimab have shown efficacy in combination with nivolumab in melanoma treatment. Mechanistic understanding of these pathways reveals overlapping yet distinct immune regulatory circuits, necessitating individualized combination regimens (Fig. 2)[30].

#### PD-1/PD-L1 pathway

The PD-1 and its ligand PD-L1 pathway represent one of the most studied immune checkpoint mechanisms in cancer immunology. PD-1 is an inhibitory receptor expressed on activated T cells, B cells, and NK cells, whereas PD-L1 is often expressed on tumor cells, as well as other cells in the TME, including DCs, macrophages, and endothelial cells. The interaction between PD-1 and PD-L1 leads to the suppression of T cell activation, proliferation, and cytokine production, effectively dampening immune responses against the tumor. In cancer, the upregulation of PD-L1 on tumor cells allows them to engage PD-1 on T cells, resulting in T cell exhaustion and impaired anti-tumor immunity. This immune evasion mechanism is particularly effective in protecting tumors from immune surveillance. Immune checkpoint inhibitors targeting PD-1 (e.g. pembrolizumab, nivolumab) and PD-L1 (e.g. atezolizumab, durvalumab) have shown significant clinical benefits in various cancers, including melanoma, NSCLC, and renal cell carcinoma. These therapies work by blocking the PD-1/PD-L1 interaction, thereby rejuvenating exhausted T cells and restoring the immune system’s ability to target and eliminate tumor cells^[31,32]^.

#### CTLA-4 pathway

CTLA-4 is another key immune checkpoint receptor that plays a critical role in regulating T cell activity. CTLA-4 is structurally similar to CD28, a co-stimulatory receptor, but instead of providing activating signals, it delivers inhibitory signals that suppress T cell activation. CTLA-4 is primarily expressed on T cells, particularly on activated T cells and Tregs. The primary function of CTLA-4 is to downregulate immune responses during the early phases of immune activation by competing with CD28 for binding to co-stimulatory molecules (CD80/CD86) on APCs[33]. In cancer, tumors often exploit the CTLA-4 pathway to inhibit the immune response and prevent T cell activation. By enhancing the activity of CTLA-4, tumors promote immune tolerance and dampen the body’s ability to mount an effective anti-tumor response. Ipilimumab, a monoclonal antibody targeting CTLA-4, was the first immune checkpoint inhibitor approved for cancer treatment and has shown significant clinical benefits in melanoma. This therapy works by blocking the interaction between CTLA-4 and its ligands, thereby enhancing T cell activation and promoting anti-tumor immunity. The combination of anti-CTLA-4 and anti-PD-1 therapies is also being explored as a potent therapeutic strategy to achieve more durable responses in patients with advanced cancers[34].

#### Other emerging immune checkpoints

In addition to PD-1/PD-L1 and CTLA-4, several other immune checkpoints are being studied for their role in tumor immune evasion. These include T cell immunoglobulin and mucin-domain containing-3 (TIM-3), lymphocyte-activation gene 3 (LAG-3), and T cell immunoreceptor with Ig and ITIM domains (TIGIT), all of which have been implicated in regulating immune responses and promoting immune evasion in cancer. TIM-3 is expressed on various immune cells, including exhausted T cells, and its interaction with its ligand Gal-9 inhibits T cell function and promotes tumor immune escape. LAG-3, which shares similarities with CD4, is another inhibitory receptor expressed on activated T cells and Tregs. Its engagement with its ligand, MHC class II molecules, leads to T cell dysfunction and impaired immune responses. TIGIT, expressed on T cells and NK cells, interacts with its ligand, CD155, on tumor cells and APCs, inhibiting immune cell activation and promoting immune suppression in the TME[35]. Therapies targeting these immune checkpoints are currently under investigation, with several monoclonal antibodies being developed to block the interaction between these molecules and their ligands[35].

#### Immune checkpoint resistance and mechanisms of escape

While immune checkpoint inhibitors have revolutionized cancer therapy, not all patients respond to these treatments, and some tumors can acquire resistance to these therapies. Several mechanisms have been identified that contribute to resistance, including mutations in the tumor’s antigen presentation machinery, which impair the recognition of tumor antigens by immune cells. For example, downregulation or loss of MHC class I molecules on tumor cells can prevent T cell recognition and reduce the effectiveness of PD-1/PD-L1 blockade[36]. Additionally, the presence of an immunosuppressive TME, with high levels of Tregs, MDSCs, and inhibitory cytokines such as TGF-β, can limit the efficacy of immune checkpoint blockade therapies. Tumors may also adapt by upregulating alternative immune checkpoints or modifying their immune signature to evade immune attacks, thus necessitating the development of combination therapies that target multiple immune checkpoints and address the heterogeneous nature of the TME[37].

#### Combining immune checkpoint blockade with other therapies

To overcome immune resistance and enhance the efficacy of immune checkpoint inhibitors, combination therapies are being explored. These include combining checkpoint inhibitors with other immunotherapies, such as cancer vaccines, adoptive T cell therapies, and cytokine-based treatments like interleukin-2. These combinations aim to boost immune cell activation, enhance antigen presentation, and promote a more favorable TME for immune activity. Furthermore, combining immune checkpoint blockade with conventional therapies, such as chemotherapy, radiation, or targeted therapies, has shown promise in preclinical models and early-phase clinical trials. These treatments can induce tumor cell death, release tumor antigens, and increase the infiltration of immune cells into the tumor, thus enhancing the effectiveness of immune checkpoint inhibitors^[38,39]^.

### The TME

**Figure 3.**
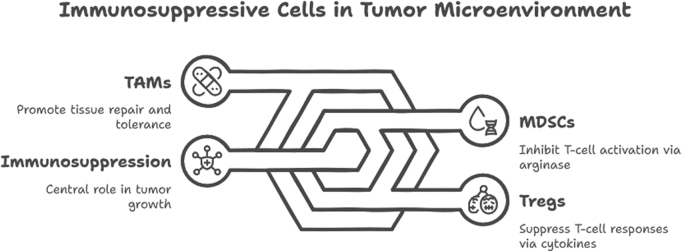
Tumor microenvironment dynamics.

The TME is a dynamic and complex ecosystem composed not only of malignant cells, but also of stromal components, immune cells, blood vessels, extracellular matrix (ECM), and a wide array of cytokines and growth factors. This environment plays a pivotal role in shaping tumor progression, immune evasion, metastasis, and response to therapy. Far from being a passive bystander, the TME actively supports tumor survival and modulates the function of infiltrating immune cells, often skewing the immune response toward tolerance or suppression (Fig. 3)^[40,41]^. One of the hallmark features of the TME is immune cell heterogeneity. While anti-tumor immune effectors such as cytotoxic CD8^+^ T cells and NK cells may infiltrate the tumor, their activity is frequently suppressed or exhausted by regulatory elements within the microenvironment. Immunosuppressive cells – including Tregs, MDSCs, and TAMs – accumulate in the TME and secrete inhibitory cytokines such as TGF-β and IL-10, dampening effective immune responses[42].

Hypoxia is another defining characteristic of the TME, resulting from disorganized and insufficient vasculature. Hypoxic conditions not only promote genetic instability and angiogenesis but also alter immune cell metabolism and function. Hypoxia-inducible factors can upregulate immune checkpoint molecules like PD-L1 on tumor and stromal cells, contributing to immune evasion. Furthermore, the metabolic competition within the TME – characterized by glucose depletion, lactic acid accumulation, and nutrient restriction – impairs effector T cell function and supports the survival of immunosuppressive cells[43]. The ECM, traditionally viewed as structural support, is now recognized as a key modulator of immune cell trafficking and activation. Tumor-associated fibroblasts remodel the ECM and secrete chemokines and matrix metalloproteinases that create physical and biochemical barriers to immune infiltration. Additionally, they can induce a tolerogenic phenotype in DCs and promote Treg expansion, further fostering an immune-permissive niche[44].

Angiogenesis, driven by factors such as VEGF, contributes to both tumor expansion and immune regulation. The newly formed tumor vasculature is often abnormal and leaky, creating regions of low perfusion and high interstitial pressure that impede immune cell access. VEGF also exerts direct immunosuppressive effects by inhibiting DC maturation and promoting MDSC recruitment[45]. Importantly, the interactions within the TME are bidirectional. Immune cells can influence tumor behavior and the composition of the stroma. For example, chronic inflammation mediated by tumor-associated neutrophils and macrophages can promote genomic instability, epithelial-to-mesenchymal transition, and resistance to therapy[46].

### Immunotherapy in cancer

Immunotherapy has revolutionized cancer treatment by harnessing the patient’s immune system to recognize and eliminate tumor cells more effectively. Unlike traditional therapies such as chemotherapy and radiation, which directly target tumor cells but often cause systemic toxicity, immunotherapy seeks to restore or enhance the immune system’s natural capacity for tumor control. This paradigm shift has opened new avenues for durable responses and long-term survival in various malignancies (Fig. 3)^[47–49]^. One of the most transformative advances in cancer immunotherapy is the development of immune checkpoint inhibitors (ICIs). These agents target inhibitory receptors such as PD-1, its ligand PD-L1, and CTLA-4, which tumors exploit to suppress T cell activation. By blocking these checkpoints, ICIs reinvigorate exhausted T cells, restore cytotoxic function, and promote tumor clearance. Clinical trials have demonstrated significant survival benefits in cancers including melanoma, NSCLC, renal cell carcinoma, and more recently, cervical and head and neck cancers. For example, pembrolizumab, a PD-1 inhibitor, has improved 5-year survival rates in advanced melanoma from less than 20% to approximately 34%, marking a substantial therapeutic milestone (Tables 1 and 2)^[50–53]^.

**Table 1:** Landmark clinical trials of PD-1/PD-L1 inhibitors

Clinical trial	Tumor type	Therapy	Phase	Response rate	Key findings
KEYNOTE-001	Non-small cell lung cancer (NSCLC)	Pembrolizumab (Anti-PD-1)	I/II	41.3% (overall response)	First phase I/II trial to demonstrate pembrolizumab’s efficacy in NSCLC
CheckMate-017	Non-small cell lung cancer (NSCLC)	Nivolumab (Anti-PD-1)	III	20% (overall response in second-line)	Showed significant survival benefit compared to docetaxel in advanced squamous NSCLC
IMpower150	Non-small cell lung cancer (NSCLC)	Atezolizumab (Anti-PD-L1)	III	33% (combination therapy)	Demonstrated superior progression-free survival when combined with chemotherapy
KEYNOTE-024	Non-small cell lung cancer (NSCLC)	Pembrolizumab (Anti-PD-1)	III	44.8% (first-line therapy)	Pembrolizumab significantly improved overall survival compared to chemotherapy in advanced NSCLC

**Table 2:** Landmark clinical trials of CTLA-4 inhibitors

Clinical trial	Tumor type	Therapy	Phase	Response rate	Key findings
CheckMate-067	Melanoma	Ipilimumab (anti-CTLA-4)	III	58% (combination therapy)	First to demonstrate overall survival benefit with combined ipilimumab and nivolumab in advanced melanoma
CA184-024	Melanoma	Ipilimumab (anti-CTLA-4)	III	11% (monotherapy)	Showed overall survival advantage over chemotherapy
ECOG-1609	Non-small cell lung cancer	Ipilimumab (anti-CTLA-4)	III	13.2% (first-line therapy)	Ipilimumab in combination with chemotherapy in NSCLC had limited efficacy compared to monotherapy
CheckMate-568	Non-small cell lung cancer	Ipilimumab + Nivolumab (combination)	III	39% (combination therapy)	Demonstrated a higher response rate and survival benefit compared to chemotherapy

Beyond checkpoint blockade, other immunotherapeutic strategies include cancer vaccines, adoptive cell therapies (ACT), and oncolytic viruses. Cancer vaccines aim to stimulate tumor-specific immunity by presenting TAAs to the immune system, though their clinical success has been limited to date. ACT involves the *ex vivo* expansion and reinfusion of autologous tumor-reactive lymphocytes, such as chimeric antigen receptor (CAR) T cells, which have shown remarkable efficacy in hematologic malignancies like acute lymphoblastic leukemia. Oncolytic viruses selectively infect and lyse tumor cells while also eliciting systemic anti-tumor immune responses (Table 3)^[54–56]^. Combination therapies are gaining traction to overcome resistance mechanisms inherent to monotherapies. These include pairing ICIs with chemotherapy, radiation, targeted agents, or other immunomodulatory drugs, aiming to enhance tumor antigen release, increase immune infiltration, and counteract immunosuppressive pathways. For example, the combination of atezolizumab (an anti-PD-L1 antibody) with chemotherapy has become a standard first-line treatment for extensive-stage small cell lung cancer, showing improved overall survival (OS) compared to chemotherapy alone (Table 4) ^[57,58]^.

**Table 3:** Landmark clinical trials of CAR-T cell therapies

Clinical trial	Tumor type	Therapy	Phase	Response rate	Key findings
JULIET (Kymriah)	Diffuse large B-cell lymphoma (DLBCL)	Tisagenlecleucel (CAR-T)	II	52% (overall response)	First large-scale trial to show significant efficacy of CAR-T in DLBCL
ZUMA-1	Large B-cell lymphoma	Axicabtagene ciloleucel (CAR-T)	II	82% (overall response)	Demonstrated a high overall response rate in refractory DLBCL, with durable remissions
TRANSCEND NHL 001	Large B-cell lymphoma	Lisocabtagene maraleucel (CAR-T)	II	73% (overall response)	Shown to provide significant efficacy in refractory DLBCL with manageable safety profile
ELIANA	Acute lymphoblastic leukemia (ALL)	Tisagenlecleucel (CAR-T)	II	83% (overall response)	Demonstrated high complete remission rates in pediatric and young adult ALL patients

**Table 4:** Landmark clinical trials of oncolytic viruses

Clinical trial	Tumor type	Therapy	Phase	Response rate	Key findings
ONC-201	Solid tumors (varied)	ONC-201 (oncolytic virus)	I/II	40%–60% (varied response)	First-in-human trial demonstrating the safety and potential of ONC-201 in solid tumors
T-VEC	Melanoma	Talimogene laherparepvec (T-VEC)	III	16%–25% (response)	First FDA-approved oncolytic virus therapy for melanoma, demonstrating tumor regression in advanced cases
NCT02234939	Pancreatic cancer	Oncolytic herpes simplex virus (HSV)	I/II	28% (local response)	Safety and efficacy data suggest oncolytic HSV can induce partial responses in pancreatic cancer
NCT03256306	Ovarian cancer	Oncolytic adenovirus (OBP-301)	I/II	38% (local tumor control)	Showed feasibility and tumor control benefits with a manageable safety profile

### Challenges in cancer immunotherapy

While cancer immunotherapy has made significant strides in transforming cancer treatment, there are numerous challenges that hinder its widespread success. These obstacles are multifaceted, involving complexities within tumor biology, the immune system, and the clinical application of immunotherapeutic approaches[59].

#### Tumor heterogeneity

One of the most significant challenges in cancer immunotherapy is tumor heterogeneity. Tumors are composed of a diverse range of cell populations with varying genetic mutations, protein expressions, and immune evasion mechanisms. This diversity complicates the identification of universal targets for immunotherapy and can lead to partial responses or treatment resistance. Some tumor cells may not express the antigens targeted by therapies, or they may mutate rapidly, leading to the development of resistance over time[60].

#### Immune evasion and the TME

TME plays a critical role in facilitating immune evasion by tumors. The TME is composed of various immune cells, stromal cells, and signaling molecules that can create an immunosuppressive environment, hindering the ability of immune cells to effectively recognize and attack tumor cells. TAMs, MDSCs, Tregs, and tumor-associated neutrophils are among the key immune cells that contribute to immune suppression in the TME. These cells can secrete cytokines, growth factors, and ECM components that inhibit T cell activation and promote tumor growth. As a result, even when ICIs or other immunotherapies are applied, the TME may limit the therapeutic efficacy, leading to treatment resistance or relapse[61].

#### Immune-related adverse events

While immunotherapy has shown remarkable efficacy in some cancers, it can also cause immune-related adverse events (irAEs) due to the activation of the immune system against normal, healthy tissues. ICIs, CAR-T cell therapy, and other immunotherapies can cause a hyperactivation of the immune system, leading to inflammation and damage in various organs, including the skin, liver, lungs, and gastrointestinal tract. These adverse events can range from mild to severe and may require the interruption or discontinuation of treatment[62].

#### Limited response in solid tumors

While immunotherapy has achieved groundbreaking success in hematological cancers, such as leukemia and lymphoma, its efficacy in treating solid tumors remains a major challenge. Solid tumors, such as those found in breast, lung, and colorectal cancers, are often characterized by a dense stromal matrix, a lack of immunogenicity, and an immunosuppressive TME. Additionally, tumors in these settings may be less responsive to ICI or CAR-T cell therapy due to poor T cell infiltration or the presence of physical barriers that prevent immune cells from accessing the tumor. The low mutational burden of certain solid tumors also means fewer neoantigens are available for immune recognition, limiting the effectiveness of immunotherapies[63].

#### Resistance to immunotherapy

Despite initial responses to immunotherapy, many patients experience relapse or resistance to treatment. Resistance mechanisms can be both intrinsic and acquired. Intrinsic resistance refers to the tumor’s ability to evade immune detection from the outset, often due to low antigen expression or a lack of immune cell infiltration. Acquired resistance, on the other hand, occurs after an initial period of response, where tumors adapt to evade immune surveillance by altering their immune landscape. For example, tumor cells may upregulate alternative immune checkpoints, such as TIM-3 or LAG-3, to counteract the effects of PD-1/PD-L1 inhibition^[64,65]^

#### Cost and accessibility

Another significant challenge in cancer immunotherapy is the high cost and limited accessibility of treatment. Immunotherapies, particularly CAR-T cell therapies and ICIs, are often prohibitively expensive, with the cost of a single treatment regimen reaching hundreds of thousands of dollars. This high cost poses a barrier to access for many patients, particularly those in low- and middle-income countries. Additionally, the complex production process for treatments like CAR-T cells, which involves harvesting and genetically modifying a patient’s own T cells, can further complicate the logistics of providing these therapies to a wide patient population[66].

#### Identifying predictive biomarkers

One of the most significant challenges in cancer immunotherapy is determining which patients will benefit from treatment. The success of immunotherapy is often unpredictable, with some patients experiencing durable responses while others show no benefit. Predictive biomarkers are needed to identify patients who are most likely to respond to immunotherapy, allowing for personalized treatment strategies. For example, the presence of PD-L1 expression or high tumor mutational burden (TMB) can predict response to ICIs. However, these biomarkers are not universally applicable, and their utility in guiding treatment decisions is still under investigation[67].

#### Immunotherapy in pediatric and elderly populations

The use of immunotherapy in pediatric and elderly populations presents unique challenges. Pediatric cancers are biologically distinct from adult cancers and may not respond to immunotherapy in the same way. The immune system in children is still developing, which can influence the efficacy of immunotherapies. Moreover, some pediatric cancers, such as neuroblastoma, may be less immunogenic, making them more challenging targets for immunotherapy. In elderly patients, age-related immune system dysfunction, including immunosenescence and comorbidities, can affect the response to immunotherapy[68].

#### Tumor antigen identification

**Figure 4.**
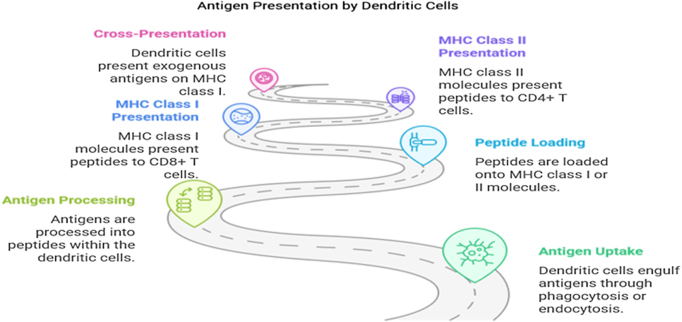
Antigen presentation mechanisms.

For immunotherapies like cancer vaccines and CAR-T cell therapy to be effective, they must target specific TAAs that are expressed on the surface of cancer cells. However, the identification of reliable and tumor-specific antigens has proven to be a major challenge. Many of the antigens targeted by immunotherapies are also expressed on normal cells to some degree, leading to the risk of off-target effects and damage to healthy tissues. Additionally, tumors can evolve and downregulate the expression of these antigens, limiting the effectiveness of therapies that rely on antigen recognition (Fig. 4)[69].

#### Combination therapies and synergistic approaches

While monotherapies with ICIs have demonstrated remarkable success in certain cancers, their efficacy remains limited in many tumor types due to primary or acquired resistance. As a result, combination strategies have emerged as a key avenue to enhance antitumor immunity, overcome immunosuppressive barriers, and improve clinical outcomes.

#### Checkpoint inhibitors plus chemotherapy

Combining ICIs with conventional chemotherapy has become a widely studied and clinically validated strategy. Chemotherapy can induce immunogenic cell death, increasing neoantigen presentation and priming of cytotoxic T cells. For example, the KEYNOTE-189 trial demonstrated that combining pembrolizumab with platinum-based chemotherapy significantly improved OS in patients with non-squamous NSCLC, with a median OS of 22.0 months versus 10.7 months with chemotherapy alone. This approach has since become a standard of care in multiple cancers[70].

#### Checkpoint inhibitors plus radiotherapy

Radiation therapy enhances antigen release and modulates the TME, creating conditions that are conducive to T cell infiltration. The phenomenon known as the abscopal effect – tumor regression at sites distant from the irradiated field – has been increasingly observed in patients receiving concurrent ICIs and radiotherapy. Ongoing trials, such as NRG-GY017 in cervical cancer and PACIFIC in NSCLC, continue to evaluate the synergy between radiation and immunotherapy[69].

#### Checkpoint inhibitors plus targeted therapy

Combining ICIs with targeted agents can modulate the immunosuppressive tumor milieu. In renal cell carcinoma (RCC), the combination of nivolumab (PD-1 inhibitor) and cabozantinib (VEGFR inhibitor) significantly improved progression-free survival and OS compared to sunitinib monotherapy in the CheckMate-9ER trial. These results highlight the potential of combining immunotherapy with angiogenesis inhibitors to reshape the tumor vasculature and immune accessibility[70].

#### Oncolytic viruses and immunotherapy

Oncolytic viruses selectively infect and lyse tumor cells, releasing TAAs and stimulating an immune response. The most prominent example is Talimogene laherparepvec, an oncolytic HSV-1 virus, which in combination with pembrolizumab showed improved objective response rates in advanced melanoma compared to either therapy alone. This approach is currently being expanded to other tumor types[70].

#### Emerging multimodal approaches

Recent innovations are exploring triple therapies that integrate checkpoint blockade with chemotherapy and targeted therapies or with personalized cancer vaccines. Additionally, adoptive cell transfer (e.g. CAR-T cells) and bispecific antibodies are being investigated in combination with ICIs to enhance T cell persistence and tumor targeting. While these strategies vary in efficacy depending on tumor type and immune contexture, the most promising combinations to date involve checkpoint inhibitors with chemotherapy (NSCLC, triple-negative breast cancer) and targeted agents (RCC, hepatocellular carcinoma). The success of these approaches underscores the necessity of tailoring therapy to tumor-specific immune evasion mechanisms and biomarker-driven patient selection.

### NF-κB pathway in tumor immunosuppression and targeted therapeutic strategies

The NF-κB signaling pathway plays a central role in regulating immune responses, inflammation, cell proliferation, and survival. In the context of cancer, constitutive or aberrant activation of NF-κB contributes significantly to the establishment of an immunosuppressive TME, promoting tumor progression and resistance to immunotherapy[71].

### Role of NF-κB in tumor-associated inflammation and immune evasion

NF-κB acts as a transcriptional regulator of numerous pro-inflammatory cytokines (e.g. IL-6, TNF-α, IL-1β), chemokines (e.g. CCL2, CXCL8), and survival factors that collectively enhance tumor cell proliferation, angiogenesis, and immune evasion. Within the TME, NF-κB activation in both cancer and stromal cells facilitates the recruitment of immunosuppressive cells such as Tregs, MDSCs, and TAMs with an M2-like phenotype. This immune landscape impairs cytotoxic T cell activity and fosters tumor tolerance. Moreover, NF-κB can be activated by various upstream oncogenic signals, including those from Toll-like receptors, TNF receptor family members, and pattern recognition receptors (PRRs), thereby creating a self-reinforcing inflammatory loop within the TME[72].

### Therapeutic strategies targeting NF-κB signaling

Given its multifaceted role in tumor-promoting inflammation and immune suppression, NF-κB has emerged as a promising therapeutic target. Several strategies have been developed to inhibit this pathway:

#### IκB kinase inhibitors

IKKα and IKKβ are essential mediators of the canonical NF-κB pathway, leading to phosphorylation and degradation of the inhibitor IκBα. Small molecule inhibitors targeting IκB kinases (IKKs) – such as BMS-345541 and MLN120B – have shown preclinical efficacy in reducing tumor-associated cytokine production and enhancing antitumor immunity. These agents suppress NF-κB-dependent gene expression and re-sensitize tumors to apoptosis and immune-mediated killing[73].

#### Proteasome inhibition

The 26S proteasome is required for IκBα degradation and subsequent NF-κB nuclear translocation. Bortezomib, a clinically approved proteasome inhibitor for multiple myeloma, effectively blocks NF-κB activation and induces apoptosis in malignant cells. Beyond direct cytotoxic effects, bortezomib has been shown to modulate immune responses by reducing immunosuppressive cytokine production and enhancing DC function[74].

#### Natural and synthetic compounds

Curcumin, parthenolide, and synthetic analogs like BAY 11-7082 have demonstrated the ability to inhibit NF-κB activity and attenuate inflammatory signaling in tumor models. These agents are under investigation for use in combination with immunotherapy to augment T cell responses and reverse TME-mediated resistance[74].

### Future directions in cancer immunotherapy

The integration of artificial intelligence (AI) and machine learning (ML) is rapidly transforming cancer immunotherapy. AI-driven algorithms can mine complex genomic, transcriptomic, and clinical datasets to predict responses to immunotherapy, identify new biomarkers, and stratify patients. Transfer learning and deep learning models are particularly valuable in rare tumor types with limited datasets. Platforms like DeepTCR and ImmuNet are pioneering this space. In addition, digital pathology and image-based AI can identify subtle patterns of immune infiltration predictive of treatment response. Incorporating these technologies into clinical practice will enhance precision oncology and treatment personalization.

#### Personalized immunotherapy approaches

One of the most exciting future directions in cancer immunotherapy is the shift toward personalized treatment strategies. With advances in genomic and proteomic profiling, researchers are gaining a deeper understanding of the unique molecular landscape of each patient’s tumor. Personalized immunotherapies will focus on tailoring treatments based on an individual’s genetic mutations, tumor antigens, and immune profile. For instance, precision medicine could involve identifying specific tumor neoantigens that are unique to each patient and designing individualized cancer vaccines or adoptive T cell therapies[75].

#### Overcoming tumor heterogeneity and antigen escape

Tumor heterogeneity remains a significant challenge to the success of immunotherapy, as tumors often consist of a variety of subpopulations of cells with different mutations and immune evasion mechanisms. Future research will likely focus on developing therapies that can target a broad range of tumor cells, including those that may be resistant to existing treatments. One promising avenue is the use of multi-target therapies, which aim to simultaneously target multiple antigens or signaling pathways to overcome tumor diversity and reduce the likelihood of antigen escape. Moreover, advancements in single-cell technologies, such as single-cell RNA sequencing, are providing valuable insights into TMEs and identifying new TAAs that could be targeted by immunotherapy^[76,77]^.

#### Enhanced TME modulation

The TME plays a crucial role in mediating immune resistance, and overcoming the immunosuppressive nature of the TME is key to improving immunotherapy outcomes. Future therapeutic strategies may involve strategies that reprogram the TME to become more conducive to anti-tumor immunity. This could include the development of drugs or biologics that deplete or inhibit immunosuppressive cells, such as Tregs, TAMs, and MDSCs. Additionally, therapies that target immune checkpoints within the TME, such as LAG-3 or TIM-3, are gaining interest as a way to further enhance immune responses. Combining ICIs with therapies that alter the TME could provide a more comprehensive and effective treatment approach[78].

#### Combination immunotherapy strategies

Combining multiple immunotherapies or pairing immunotherapy with other treatment modalities, such as chemotherapy, targeted therapy, or radiation, is expected to be a major focus of future cancer immunotherapy research. Combination strategies aim to enhance immune activation, overcome resistance mechanisms, and increase the breadth of treatment efficacy. For example, combining ICIs with targeted therapies can synergize by both increasing immune responses and directly targeting tumor cells. Additionally, combining immunotherapy with radiation or chemotherapy could stimulate immune responses through tumor cell death, thus providing an additional means to activate the immune system[79].

#### Expanding immunotherapy to solid tumors

While immunotherapy has had significant success in treating hematologic cancers, its efficacy in solid tumors remains a challenge. Solid tumors, including lung, breast, and colorectal cancers, have been less responsive to ICIs and other immunotherapies due to factors such as poor immune cell infiltration, low TMB, and the presence of physical barriers like a dense stromal matrix. Future research will likely focus on improving immune cell penetration into solid tumors, enhancing tumor antigen recognition, and modulating the TME to be more immunogenic[80].

#### Overcoming irAE

While immunotherapy has demonstrated impressive clinical benefits, irAEs remain a significant concern. These adverse events occur when the immune system attacks healthy tissues, leading to inflammation and damage in various organs. As immunotherapies become more widely used, understanding the mechanisms underlying irAEs and developing strategies to mitigate these effects will be a critical focus. Future research may explore the use of biomarkers to predict patients who are at higher risk for developing irAEs, as well as the development of strategies to manage or prevent these adverse events without compromising the therapeutic benefits of immunotherapy[81].

#### Investigating the role of the microbiome in immunotherapy

Recent research has highlighted the significant role that the microbiome – the community of microorganisms living in and on the body – plays in modulating immune responses. Emerging evidence suggests that the gut microbiome, in particular, can influence the effectiveness of immunotherapy, including ICIs. By interacting with the immune system, the microbiome can enhance immune responses and potentially improve the efficacy of immunotherapy[82].

#### AI and ML in immunotherapy

Advances in AI and ML are poised to revolutionize cancer immunotherapy by improving the identification of predictive biomarkers, optimizing treatment regimens, and aiding in the design of new therapies. AI can be used to analyze large-scale genomic and clinical data, identify patterns in patient responses, and predict which patients are most likely to benefit from specific immunotherapies. ML algorithms can also assist in drug discovery by identifying new immune modulators or potential targets for therapy. These technologies hold great promise for accelerating the development of personalized and effective immunotherapeutic strategies[83].

### Cancer vaccines and neoantigen-based therapies

Cancer vaccines that target tumor-specific neoantigens are an area of great interest for future immunotherapy development. Neoantigens, which arise from mutations in tumor cells, are unique to each individual tumor and can serve as highly specific targets for immune recognition. Personalized cancer vaccines that incorporate these neoantigens are being investigated as a way to stimulate a patient’s immune system to specifically target and destroy tumor cells. Additionally, neoantigen-based therapies, such as neoantigen-directed T cell therapies, are being explored as next-generation immunotherapies^[84,85]^.

#### Enhancing global access to immunotherapy

As immunotherapy continues to advance, a key challenge will be ensuring that these promising treatments are accessible to patients around the world. The high cost of immunotherapy and the complex infrastructure required for its administration limit its availability, particularly in low- and middle-income countries. Future efforts will need to focus on reducing the cost of immunotherapy, improving the manufacturing process for treatments like CAR-T cells, and exploring alternative delivery models, such as off-the-shelf therapies ^[86–88]^.

## Conclusion

Cancer immunotherapy represents one of the most significant advances in the field of oncology, offering promising treatment options that harness the body’s immune system to target and eliminate cancer cells. Over the past few decades, immunotherapies such as ICIs, CAR-T cell therapy, and cancer vaccines have demonstrated remarkable clinical success, especially in cancers previously considered resistant to conventional therapies. However, despite these advancements, challenges remain, including tumor heterogeneity, immune evasion, adverse effects, and limited efficacy in certain cancer types. Future research and clinical trials hold great promise for overcoming these challenges. Personalized immunotherapy, which tailors treatments to individual patients based on their genetic, molecular, and immune profiles, is expected to enhance the effectiveness of current therapies. Additionally, combining immunotherapy with other treatment modalities and improving the TME are key areas of ongoing exploration. The integration of AI, deeper understanding of the microbiome’s role, and novel strategies for improving access to immunotherapy are also critical steps toward improving outcomes and making these therapies more widely available.

## Data Availability

Not applicable as this is a narrative review.

## Research registration unique identifying number (UIN)

Not applicable as this is a narrative review.

## References

[1] Marzagalli M Ebelt ND Manuel ER. Unraveling the crosstalk between melanoma and immune cells in the tumor microenvironment. Semin Cancer Biol 2019;59:236–50. 10.1016/j.semcancer.2019.08.002 31404607

[2] Uchi H Stan R Turk MJ. Unraveling the complex relationship between cancer immunity and autoimmunity: lessons from melanoma and vitiligo. Adv Immunol 2006;90:215–41. 16730265 10.1016/S0065-2776(06)90006-6

[3] Soussan S Pupier G Cremer I. Unraveling the complex interplay between anti-tumor immune response and autoimmunity mediated by B cells and autoantibodies in the era of anti-checkpoint monoclonal antibody therapies. Front Immunol 2024;15:1343020. 38318190 10.3389/fimmu.2024.1343020 PMC10838986

[4] El-Tanani M Rabbani SA Babiker R. Unraveling the tumor microenvironment: insights into cancer metastasis and therapeutic strategies. Cancer Lett 2024;591:216894. 38626856 10.1016/j.canlet.2024.216894

[5] Fang J Lu Y Zheng J. Exploring the crosstalk between endothelial cells, immune cells, and immune checkpoints in the tumor microenvironment: new insights and therapeutic implications. Cell Death Dis 2023;14:586. 37666809 10.1038/s41419-023-06119-x PMC10477350

[6] He R Huang S Lu J. Unveiling the immune symphony: decoding colorectal cancer metastasis through immune interactions. Front Immunol 2024;15:1362709. 38415252 10.3389/fimmu.2024.1362709 PMC10897008

[7] Klein Geltink RI Kyle RL Pearce EL. Unraveling the complex interplay between T cell metabolism and function. Annu Rev Immunol 2018;36:461–88. 29677474 10.1146/annurev-immunol-042617-053019 PMC6323527

[8] Awasthi D Sarode A. Neutrophils at the crossroads: unraveling the multifaceted role in the tumor microenvironment. Int J Mol Sci 2024;25:2929. 38474175 10.3390/ijms25052929 PMC10932322

[9] Sethi N Kang Y. Unravelling the complexity of metastasis—molecular understanding and targeted therapies. Nat Rev Cancer 2011;11:735–48. 21941285 10.1038/nrc3125 PMC10961136

[10] Wang K Wang X Song L. Unraveling the complex role of neutrophils in lymphoma: from pathogenesis to therapeutic approaches. Mol Clin Oncol 2024;21:85. 39347476 10.3892/mco.2024.2783 PMC11428085

[11] Jiménez-Sánchez A Cybulska P Mager KL. Unraveling tumor–immune heterogeneity in advanced ovarian cancer uncovers immunogenic effect of chemotherapy. Nat Genet 2020;52:582–93. 32483290 10.1038/s41588-020-0630-5 PMC8353209

[12] Ning J Wang Y Tao Z. The complex role of immune cells in antigen presentation and regulation of T-cell responses in hepatocellular carcinoma: progress, challenges, and future directions. Front Immunol 2024;15:1483834. 39502703 10.3389/fimmu.2024.1483834 PMC11534672

[13] Laderach DJ Compagno D. Unraveling how tumor-derived galectins contribute to anti-cancer immunity failure. Cancers (Basel) 2021;13:4529. 34572756 10.3390/cancers13184529 PMC8469970

[14] Kotsifaki A Alevizopoulos N Dimopoulou V. Unveiling the immune microenvironment’s role in breast cancer: a glimpse into promising frontiers. Int J Mol Sci 2023;24:15332. 37895012 10.3390/ijms242015332 PMC10607694

[15] Dong W Sheng J Cui JZ. Systems immunology insights into brain metastasis. Trends Immunol 2024;45:903–16. 39443266 10.1016/j.it.2024.09.010 PMC12049182

[16] Zhang Y Chen X Hu B. Advancements in nanomedicine delivery systems: unraveling immune regulation strategies for tumor immunotherapy. Nanomedicine 2024;19:1821–40. 39011582 10.1080/17435889.2024.2374230 PMC11418288

[17] Chen GM Azzam A Ding YY. Dissecting the tumor–immune landscape in chimeric antigen receptor T-cell therapy: key challenges and opportunities for a systems immunology approach. Clin Cancer Res 2020;26:3505–13. 32127393 10.1158/1078-0432.CCR-19-3888 PMC7367708

[18] Conejo-Garcia JR Lopez-Bailon LU Anadon CM. Unraveling spontaneous humoral immune responses against human cancer: a road to novel immunotherapies. J Leukoc Biol 2024;116:919–26. 39190797 10.1093/jleuko/qiae179

[19] Li T Qiao T. Unraveling tumor microenvironment of small-cell lung cancer: Implications for immunotherapy. Semin Cancer Biol 2022;86:117–25. 10.1016/j.semcancer.2022.09.005 36183998

[20] Erra Díaz F Dantas E Geffner J. Unravelling the interplay between extracellular acidosis and immune cells. Mediators Inflamm 2018;2018:1218297. 30692870 10.1155/2018/1218297 PMC6332927

[21] Chow MT Möller A Smyth MJ. Inflammation and immune surveillance in cancer. Semin Cancer Biol 2012;22:23–32. 10.1016/j.semcancer.2011.12.004 22210181

[22] Pardoll D. Does the immune system see tumors as foreign or self? Annu Rev Immunol 2003;21:807–39. 12615893 10.1146/annurev.immunol.21.120601.141135

[23] Swann JB Smyth MJ. Immune surveillance of tumors. J Clin Invest 2007;117:1137–46. 17476343 10.1172/JCI31405 PMC1857231

[24] Ribatti D. The concept of immune surveillance against tumors: the first theories. Oncotarget 2016;8:7175. 10.18632/oncotarget.12739 PMC5351698 27764780

[25] Candeias SM Gaipl US. The immune system in cancer prevention, development and therapy. Anticancer Agents Med Chem (formerly current Medicinal Chemistry-Anti-cancer agents) 2016;16:101–07. 10.2174/1871520615666150824153523 26299661

[26] Cramer DW Finn OJ. Epidemiologic perspective on immune-surveillance in cancer. Curr Opin Immunol 2011;23:265–71. 21277761 10.1016/j.coi.2011.01.002 PMC3073666

[27] Kim R. Cancer immunoediting: from immune surveillance to immune escape. Cancer Immunother 2007;121:1–14. 10.1111/j.1365-2567.2007.02587.x PMC2265921 17386080

[28] Scouten WT Francis GL. Thyroid cancer and the immune system: a model for effective immune surveillance. Expert Rev Endocrinol Metab 2006;1:353–66. 30764074 10.1586/17446651.1.3.353

[29] Krieg C Boyman O. The role of chemokines in cancer immune surveillance by the adaptive immune system Semin Cancer Biol 2009;19:76–83. 10.1016/j.semcancer.2008.10.011 19038343

[30] Parcesepe P Giordano G Laudanna C. Cancer-associated immune resistance and evasion of immune surveillance in colorectal cancer. Gastroenterol Res Pract 2016;2016:6261721. 27006653 10.1155/2016/6261721 PMC4781955

[31] Ostrand-Rosenberg S. Immune surveillance: a balance between protumor and antitumor immunity. Curr Opin Genet Dev 2008;18:11–18. 18308558 10.1016/j.gde.2007.12.007 PMC2699403

[32] Satgé D. A tumor profile in primary immune deficiencies challenges the cancer immune surveillance concept. Front Immunol 2018;9:1149. 29881389 10.3389/fimmu.2018.01149 PMC5976747

[33] Corthay A. Does the immune system naturally protect against cancer? Front Immunol 2014;5:197. 24860567 10.3389/fimmu.2014.00197 PMC4026755

[34] Hoenicke L Zender L. Immune surveillance of senescent cells—biological significance in cancer-and non-cancer pathologies. Carcinogenesis 2012;33:1123–26. 22470164 10.1093/carcin/bgs124

[35] Hamai A Benlalam H Meslin F. Immune surveillance of human cancer: if the cytotoxic T-lymphocytes play the music, does the tumoral system call the tune? Tissue Antigens 2010;75:1–8. 20196816 10.1111/j.1399-0039.2009.01401.x

[36] Pardoll DM. The blockade of immune checkpoints in cancer immunotherapy. Nat Rev Cancer 2012;12:252–64. 22437870 10.1038/nrc3239 PMC4856023

[37] Topalian SL. Targeting immune checkpoints in cancer therapy. Jama 2017;318:1647–48. 28885639 10.1001/jama.2017.14155

[38] Karin N. Chemokines and cancer: new immune checkpoints for cancer therapy. Curr Opin Immunol 2018;51:140–45. 29579623 10.1016/j.coi.2018.03.004

[39] Gu D Ao X Yang Y. Soluble immune checkpoints in cancer: production, function and biological significance. J Immunother Cancer 2018;6:1–4. 30482248 10.1186/s40425-018-0449-0 PMC6260693

[40] Park R Winnicki M Liu E. Immune checkpoints and cancer in the immunogenomics era. Brief Funct Genomics 2019;18:133–39. 30137232 10.1093/bfgp/ely027 PMC6488970

[41] Arneth B. Tumor microenvironment. Medicina (B Aires) 2019;56:15. 10.3390/medicina56010015 PMC7023392 31906017

[42] Balkwill FR Capasso M Hagemann T. The tumor microenvironment at a glance. J Cell Sci 2012;125:5591–96. 23420197 10.1242/jcs.116392

[43] Xiao Y Yu D. Tumor microenvironment as a therapeutic target in cancer. Pharmacol Ther 2021;221:107753. 33259885 10.1016/j.pharmthera.2020.107753 PMC8084948

[44] Hinshaw DC Shevde LA. The tumor microenvironment innately modulates cancer progression. Cancer Res 2019;79:4557–66. 31350295 10.1158/0008-5472.CAN-18-3962 PMC6744958

[45] Roma-Rodrigues C Mendes R Baptista PV. Targeting tumor microenvironment for cancer therapy. Int J Mol Sci 2019;20:840. 30781344 10.3390/ijms20040840 PMC6413095

[46] Wang M Zhao J Zhang L. Role of tumor microenvironment in tumorigenesis. J Cancer 2017;8:761. 28382138 10.7150/jca.17648 PMC5381164

[47] Kirkwood JM Butterfield LH Tarhini AA. Immunotherapy of cancer in 2012. CA Cancer J Clin 2012;62:309–35. 22576456 10.3322/caac.20132 PMC3445708

[48] Ribas A Wolchok JD. Cancer immunotherapy using checkpoint blockade. Science 2018;359:1350–55. 29567705 10.1126/science.aar4060 PMC7391259

[49] Byun DJ Wolchok JD Rosenberg LM. Cancer immunotherapy—immune checkpoint blockade and associated endocrinopathies. Nat Rev Endocrinol 2017;13:195–207. 28106152 10.1038/nrendo.2016.205 PMC5629093

[50] Miliotou AN Papadopoulou LC. CAR T-cell therapy: a new era in cancer immunotherapy. Curr Pharm Biotechnol 2018;19:5–18. 29667553 10.2174/1389201019666180418095526

[51] June CH O’Connor RS Kawalekar OU. CAR T cell immunotherapy for human cancer. Science 2018;359:1361–65. 29567707 10.1126/science.aar6711

[52] Igarashi Y Sasada T. Cancer vaccines: toward the next breakthrough in cancer immunotherapy. J Immunol Res 2020;2020:5825401. 33282961 10.1155/2020/5825401 PMC7685825

[53] Naidoo J Page DB Wolchok JD. Immune modulation for cancer therapy. Br J Cancer 2014;111:2214–19. 25211661 10.1038/bjc.2014.348 PMC4264429

[54] Marhelava K Pilch Z Bajor M. Targeting negative and positive immune checkpoints with monoclonal antibodies in therapy of cancer. Cancers (Basel) 2019;11:1756. 31717326 10.3390/cancers11111756 PMC6895894

[55] Hemminki O Dos Santos JM Hemminki A. Oncolytic viruses for cancer immunotherapy. J Hematol Oncol 2020;13:1–5. 32600470 10.1186/s13045-020-00922-1 PMC7325106

[56] Chiocca EA Rabkin SD. Oncolytic viruses and their application to cancer immunotherapy. Cancer Immunol Res 2014;2:295–300. 24764576 10.1158/2326-6066.CIR-14-0015 PMC4303349

[57] Falcone I Conciatori F Bazzichetto C. Tumor microenvironment: implications in melanoma resistance to targeted therapy and immunotherapy. Cancers (Basel) 2020;12:2870. 33036192 10.3390/cancers12102870 PMC7601592

[58] Madeddu C Donisi C Liscia N. EGFR-mutated non-small cell lung cancer and resistance to immunotherapy: role of the tumor microenvironment. Int J Mol Sci 2022;23:6489. 35742933 10.3390/ijms23126489 PMC9224267

[59] Obeagu EI Obeagu GU. Breast cancer: a review of risk factors and diagnosis. Medicine (Baltimore) 2024;103:e36905. 38241592 10.1097/MD.0000000000036905 PMC10798762

[60] Obeagu EI Obeagu GU. Exploring neutrophil functionality in breast cancer progression: a review. Medicine (Baltimore) 2024;103:e37654. 38552040 10.1097/MD.0000000000037654 PMC10977563

[61] Obeagu EI Obeagu GU. Lymphocyte infiltration in breast cancer: a promising prognostic indicator. Medicine (Baltimore) 2024;103:e40845. 39654199 10.1097/MD.0000000000040845 PMC11631027

[62] Obeagu EI Obeagu GU. Breastfeeding’s protective role in alleviating breast cancer burden: a comprehensive review. Ann Med Surg (Lond) 2024;86:2805–11. 38694322 10.1097/MS9.0000000000001914 PMC11060284

[63] Obeagu EI Obeagu GU. Exploring the profound link: breastfeeding’s impact on alleviating the burden of breast cancer - A review. Medicine (Baltimore) 2024;103:e37695. 38608095 10.1097/MD.0000000000037695 PMC11018178

[64] Obeagu EI Obeagu GU. Predictive models and biomarkers for survival in stage III breast cancer: a review of clinical applications and future directions. Ann Med Surg (Lond) 2024;86:5980–87. 39359789 10.1097/MS9.0000000000002517 PMC11444610

[65] Peterson C Denlinger N Yang Y. Recent advances and challenges in cancer immunotherapy. Cancers (Basel) 2022;14:3972. 36010965 10.3390/cancers14163972 PMC9406446

[66] Green AK. Challenges in assessing the cost-effectiveness of cancer immunotherapy. JAMA Network Open 2021;4:e2034020. 33464313 10.1001/jamanetworkopen.2020.34020

[67] Spencer KR Wang J Silk AW. Biomarkers for immunotherapy: current developments and challenges. Am Soc Clin Oncol Educ Book 2016;36:e493–503. 10.1200/EDBK_160766 27249758

[68] Poropatich K Fontanarosa J Samant S. Cancer immunotherapies: are they as effective in the elderly? Drugs Aging 2017;34:567–81. 28707274 10.1007/s40266-017-0479-1

[69] Martin JD Cabral H Stylianopoulos T. Improving cancer immunotherapy using nanomedicines: progress, opportunities and challenges. Nat Rev Clin Oncol 2020;17:251–66. 32034288 10.1038/s41571-019-0308-z PMC8272676

[70] Melero I Berman DM Aznar MA. Evolving synergistic combinations of targeted immunotherapies to combat cancer. Nat Rev Cancer 2015;15:457–72. 26205340 10.1038/nrc3973

[71] Brahmer JR Rodríguez-Abreu D Robinson AG. Health-related quality-of-life results for pembrolizumab versus chemotherapy in advanced, PD-L1-positive NSCLC (KEYNOTE-024): a multicentre, international, randomised, open-label phase 3 trial. Lancet Oncol 2017;18:1600–09. 29129441 10.1016/S1470-2045(17)30690-3

[72] Reck M Rodríguez–Abreu D Robinson AG. Updated analysis of KEYNOTE-024: pembrolizumab versus platinum-based chemotherapy for advanced non–small-cell lung cancer with PD-L1 tumor proportion score of 50% or greater. J Clin Oncol 2019;37:537–46. 30620668 10.1200/JCO.18.00149

[73] Hodi FS Chiarion-Sileni V Gonzalez R. Nivolumab plus ipilimumab or nivolumab alone versus ipilimumab alone in advanced melanoma (CheckMate 067): 4-year outcomes of a multicentre, randomised, phase 3 trial. Lancet Oncol 2018;19:1480–92. 30361170 10.1016/S1470-2045(18)30700-9

[74] Weiss SA Kluger H. CheckMate-067: raising the bar for the next decade in oncology. J Clin Oncol 2021;40:111. 34855466 10.1200/JCO.21.02549 PMC8718180

[75] Ito F Chang AE. Cancer immunotherapy: current status and future directions. Surg Oncol Clin 2013;22:765–83. 10.1016/j.soc.2013.06.005 24012398

[76] Ventola CL. Cancer immunotherapy, part 3: challenges and future trends. Pharm Ther 2017;42:514. PMC5521300 28781505

[77] Lin H Yang X Ye S. Antigen escape in CAR-T cell therapy: mechanisms and overcoming strategies. Biomed Pharmacother 2024;178:117252. 39098176 10.1016/j.biopha.2024.117252

[78] De Cicco P Ercolano G Ianaro A. The new era of cancer immunotherapy: targeting myeloid-derived suppressor cells to overcome immune evasion. Front Immunol 2020;11:1680. 32849585 10.3389/fimmu.2020.01680 PMC7406792

[79] Lan H Zhang W Jin K. Modulating barriers of tumor microenvironment through nanocarrier systems for improved cancer immunotherapy: a review of current status and future perspective. Drug Deliv 2020;27:1248–62. 32865029 10.1080/10717544.2020.1809559 PMC7470050

[80] Huang W Chen JJ Xing R. Combination therapy: future directions of immunotherapy in small cell lung cancer. Transl Oncol 2021;14:100889. 33065386 10.1016/j.tranon.2020.100889 PMC7567053

[81] Guha P Heatherton KR O’Connell KP. Assessing the future of solid tumor immunotherapy. Biomedicines 2022;10:655. 35327456 10.3390/biomedicines10030655 PMC8945484

[82] Rahman MM Behl T Islam MR. Emerging management approach for the adverse events of immunotherapy of cancer. Molecules 2022;27:3798. 35744922 10.3390/molecules27123798 PMC9227460

[83] Kiousi DE Kouroutzidou AZ Neanidis K. The role of the gut Microbiome in cancer immunotherapy: current knowledge and future directions. Cancers (Basel) 2023;15:2101. 37046762 10.3390/cancers15072101 PMC10093606

[84] Gao Q Yang L Lu M. The artificial intelligence and machine learning in lung cancer immunotherapy. J Hematol Oncol 2023;16:55. 37226190 10.1186/s13045-023-01456-y PMC10207827

[85] Ahmed S Mazhar MS Shabbir MF. Neoantigen-based cancer vaccines: current innovations, challenges and future directions in personalized immunotherapy. Cancer Immunology Connect 2024;1:1–10.

[86] Emens LA Romero PJ Anderson AC. Challenges and opportunities in cancer immunotherapy: a Society for Immunotherapy of Cancer (SITC) strategic vision. J Immunother Cancer 2024;12:e009063. 38901879 10.1136/jitc-2024-009063 PMC11191773

[87] Agha RA Mathew G Rashid R. Transparency in the reporting of artificial intelligence – the TITAN guideline. Prem J Sci 2025;10:100082.

[88] Pisibon C Ouertani A Bertolotto C. Immune checkpoints in cancers: from signaling to the clinic. Cancers (Basel) 2021;13:4573. 34572799 10.3390/cancers13184573 PMC8468441

